# Preparation of Novel Flaxseed Oil/Beeswax Oleogel Systems and Its Application in the Improvement of Sodium Alginate Films

**DOI:** 10.3390/gels10010078

**Published:** 2024-01-21

**Authors:** Shan Xue, Qun Huang

**Affiliations:** 1 College of Biological Science and Technology, Minnan Normal University, Zhangzhou 363000, China; 2 Engineering Research Center of Fujian Province for Fungal Industry, Zhangzhou 363000, China; 3 Research Institute of Zhangzhou-Taiwan Leisure Food and Tea Beverage, Zhangzhou 363000, China; 4 School of Public Health, Guizhou Province Engineering Research Center of Health Food Innovative Manufacturing, The Key Laboratory of Environmental Pollution Monitoring and Disease Control of Ministry of Education, Guizhou Medical University, Guiyang 550025, China; huangqunlaoshi@126.com

**Keywords:** oleogel, edible films, comprehensive score, shelf-life of *Decapterus maruadsi*

## Abstract

The purpose of this study was to prepare a novel kind of flaxseed oil (FO)/beeswax oleogel system and apply it to improve the properties of sodium alginate films. Three single factors, namely the ratio of beeswax/FO, the addition of oleogel, and the addition of glycerol, were optimized based on the comprehensive score of film characteristics: elongation at break (EAB), tensile strength (TS), hydroxyl radical clearance (HRC), and water vapor permeability (WVP) of the film. When the ratio of beeswax/FO was 7.807%, the addition of oleogel was 4.829%, and the addition of glycerol was 31.088%, the comprehensive score of the film characteristics was maximum. Moreover, the *Decapterus maruadsi* preserved by the produced films were assessed for drip loss, pH, total volatile basic nitrogen (TVB-N), thiobarbituric acid reactive substance (TBARS), and fatty acids composition. In comparison to the control, the produced films incorporated with linseed oil/beeswax oleogel had a longer shelf-life than *Decapterus maruadsi*. In conclusion, the oleogel system prepared via linseed oil/beeswax had good stability and hydrophobicity, which can significantly improve the characteristics of the film and extend the shelf-life of *Decapterus maruadsi*.

## 1. Introduction

In recent years, with the strengthening of people’s environmental awareness, natural and degradable food packaging materials have gradually become a research hotspot. In particular, natural polymer materials (proteins, polysaccharides, lipids, or a mixture of these ingredients) have attracted people’s attention due to their biodegradability, mechanical resistance, and ability to inhibit oxygen and water [1]. The application of polysaccharide-based edible film in food provides a new opportunity for developing new food packaging. Polysaccharide edible membranes are made from a mixture of natural polysaccharides, additives (emulsifiers and plasticizers), and functional ingredients (antibacterial agents/antioxidants), with the advantages of being renewable and safe to eat [2].

Alginate is a marine-derived polysaccharide composed of *D*-mannouronic acid and *L*-gulonuronic acid in different arrangements and molecular weights. Sodium alginate comes from a wide range of sources and has the advantages of safety, nontoxicity, low cost, suitable gel properties, hydrophilicity, and so on. However, whether it is polysaccharide-based film or protein-based film, their oxygen and water barrier is not good enough. Thus, some hydrophobic/lipid substances can usually be added to the film to solve this problem. At the same time, one single lipid addition cannot achieve multiple film quality improvements. Therefore, it is necessary to add some additional mixture to the film-forming matrix to optimize the edible film to perform a number of predetermined functions, so that they can effectively replace synthetic packaging [3].

Due to the limitations of forming edible films from a single material, composite components containing a variety of materials, such as hydrophilic colloid and lipid components, are usually used to make the films [4]. The addition of oil to edible film can improve the water barrier properties, decrease its moisture absorption characteristics, reduce the surface roughness of the film, and enhance the specular reflectance of the air-film interface [4]. Although there have been some literature reports on the addition of lipids to edible films, it should be noted that the oil is hydrophobic, it easily leaks out in the film-forming process, and even oxidizes [3,4]. Thus, oleogel seems to be a good choice, which has been widely considered by scientific researchers.

Oleogel is an effective method to obtain ideal plant solid fat by adding gel factor to vegetable oil to interfere with oil crystallization. The oleogel can self-assemble to form a three-dimensional network to trap the oil, thus slowing down the oil migration. Flaxseed oil (FO) is rich in unsaturated fatty acids, especially linoleic acid and alpha-linolenic acid. Alpha-linolenic acid is an essential fatty acid for maintaining normal physiological functions and growth and development of the body and has various physiological functions, such as improving immunity, improving vision, lowering cholesterol, and preventing cardiovascular diseases [5,6]. FO is widely used in fat replacement because of its high content of n−3 fatty acids [7,8].

The addition of wax is helpful for maintaining the oleogel with good stability. Beeswax has unique texture properties, and stable oleogel with high oil binding can be obtained by adding a low concentration [9]. Although there is a progressive increase in exploring the potential applications of oleogels in food systems, no literature is available on the use of oleogels as ingredients of edible films except for studies carried out by Yousuf et al. [3], Xiao et al. [10], Li et al. [11], and Shin et al. [12].

Therefore, the study aimed to prepare a novel kind of FO/beeswax oleogel system and apply it to the improvement of the properties of sodium alginate films in order to provide an innovative basis and application reference for the research and development of new composite films.

## 2. Results and Discussion

### 2.1. Results of Single Factor 

#### 2.1.1. Effect of the Ratio of Beeswax/FO on the Elongation at Break (EAB), Tensile Strength (TS), Hydroxyl Radical Clearance (HRC), and Water Vapor Permeability (WVP) of the Film

**Figure 1:**
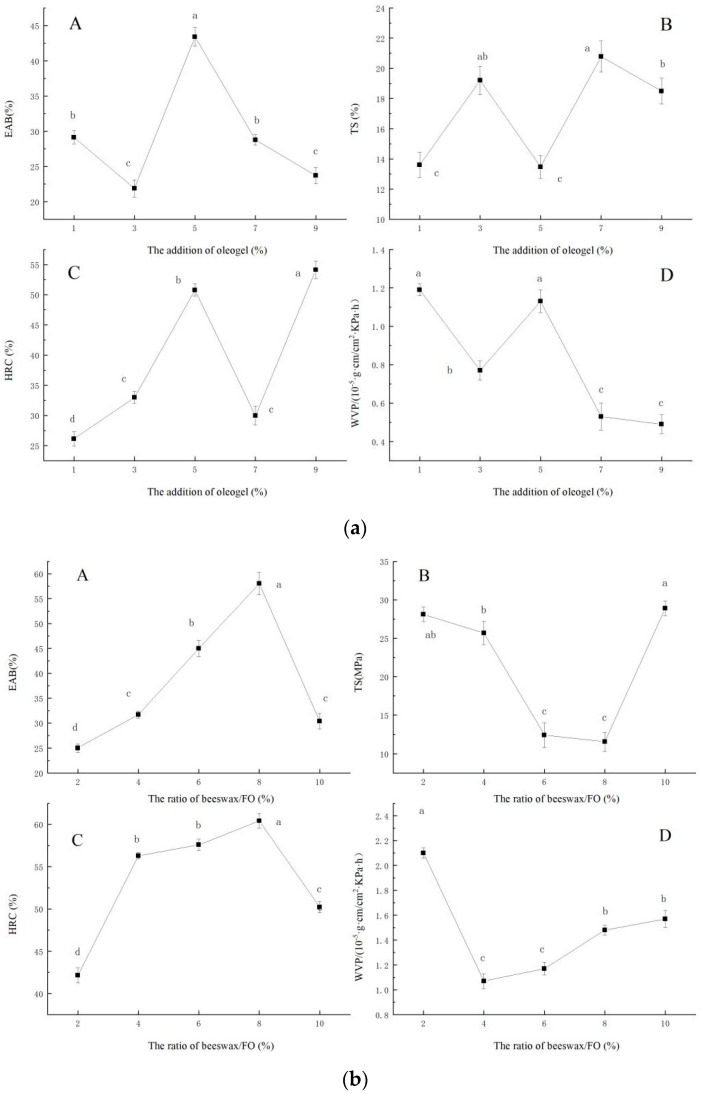

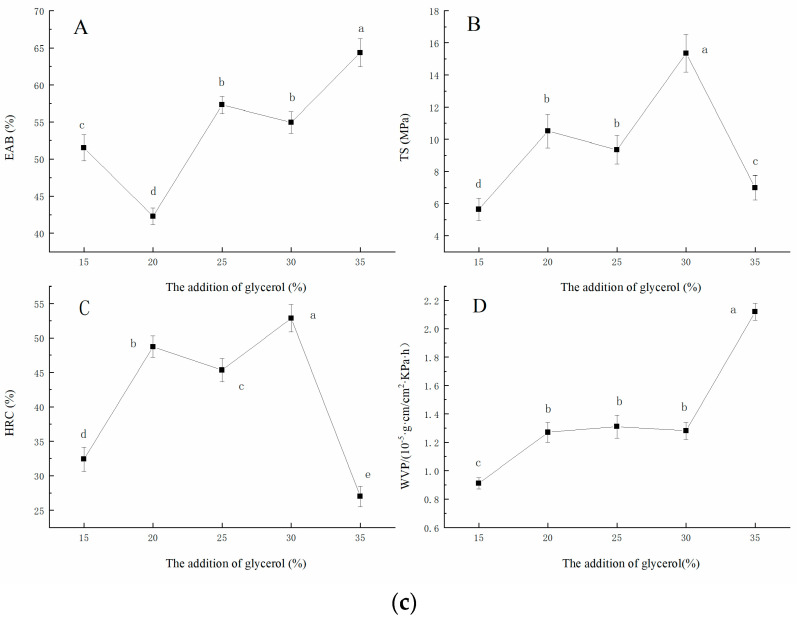
Effect of ratio of beeswax/FO (**a**), addition of oleogel (**b**), and addition of glycerol (**c**) on the EAB, TS, HRC, and WVP of the film, respectively. ((**a**): The effect of the ratio of beeswax/FO on the EAB (A), TS (B), HRC (C), and WVP (D) of the film. (**b**): The effect of the addition of oleogel on the EAB (A), TS (B), HRC (C), and WVP (D) of the film. (**c**): The effect of the addition of glycerol on the EAB (A), TS (B), HRC (C), and WVP (D) of the film.)

The effect of the ratio of beeswax/FO on the EAB (A), TS(B), HRC(C), and WVP(D) of the film were shown in Figure 1a. When the ratio of beeswax/FO was 2–10%, EAB and HRC of the films first increased and then decreased (*p* < 0.05), and TS and WVP decreased first and then increased (*p* < 0.05). When the ratio of beeswax/FO was 8%, EAB had the maximum value, TS had the lowest value, and HRC also had the maximum value. When the ratio of beeswax/FO was 4%, WVP had the minimum value, and the film had the best water vapor barrier ability. Natural waxes are often used to prepare oleogels [3]. Meng, Li, Li, and Liu [13] studied the difference between three oleogels structured by three food-grade plant waxes, namely rice bran wax, palm wax, and beeswax. They found that the beeswax-based oleogels had the highest oil-holding capacity, and the microcrystal structure was the smallest, which showed a fine acicular shape. Moreover, the chemical composition (beeswax and flaxseed oil) of the oleogel more or less interacted with sodium alginate, and different mass fractions of gels led to great differences in the crystal types of oleogel. Therefore, the addition of beeswax/FO characterized with different crystal types resulted in different properties of the films.

#### 2.1.2. Effect of the Addition of Oleogel on the EAB, TS, HRC, and WVP of the Film

As shown in Figure 1b, the addition of oleogel on the EAB (A), TS (B), HRC (C), and WVP (D) of the film is presented, respectively. The oleogel was a composite system. When the oleogel was added to the sodium alginate solution, the internal structure and mechanical properties of the film were changed. The EAB, TS, and HRC of the films did not change regularly with the increase of oleogels addition. When the oleogel content was 5%, the EAB, HRC, and WVP of the film had large values, while TS was the smallest. EAS and TS are important indicators for measuring the mechanical properties of packaging materials, and these two indicators are also important technical indicators affecting industrial applications [14]. Fabra, Talens, and Chiralt [15] reported that the addition of oil to the film matrix is believed to cause a disruption in the biopolymer network which subsequently results in an increase in flexibility and a decline in tensile strength. However, in this study, there was no obvious regular increase or decrease in EAB and TS in oleogel-containing films, which indicated that the oleogel-containing films may restrain the deterioration of biopolymer matrix of sodium alginate-based films. In addition, WVP showed an overall decreasing trend. Especially in the range of 5 to 9%, the WVP was greatly decreased. The occurrence of this phenomenon was related to the addition of oleogel, which can increase the hydrophobicity of the film. According to Zhang et al. [16], adding ginger oil to the film can reduce the WVP of the film.

#### 2.1.3. Effect of the Addition of Glycerol on EAB, TS, HRC, and WVP of the Film

The effect of the addition of glycerol on the EAB (A), TS(B), HRC(C), and WVP(D) of the films are presented in the Figure 1c. Glycerol, as a plasticizer, can break the original structure of polysaccharide polymer and form new hydrogen bonds, thus enhancing plasticity. According to Wang, Wang, Wang, and Wang [17], different plasticizers had different effects on the properties of different substrates, and the appropriate addition of glycerol can improve the mechanical and barrier properties of the film. This phenomenon may be due to the relatively small molecular weight of glycerol, which contains more hydroxyl groups so that it has a good hydrophilicity. Small molecules of plasticizer can penetrate into the internal structure of the film, forming a new intermolecular force, which makes the structure of the film become loose from dense [18]. At the same time, plasticizer is a hydrophilic agent, which affects the evaporation of water in the film, and thus cause the thickness, structure, and features to change [19].

### 2.2. Comprehensive Score Analysis of Indicator Film Properties

#### 2.2.1. Suitability Test

In this study, 3 × 3 groups of data (Table 1) were randomly selected for principal component analysis, and EAB, TS, HRC, and WVP values of the film were calculated as analysis objects.

The Kaiser-Meyer-Olkin measure of sampling adequacy was used to justify the reliability of the comprehensive score of the film properties. If the value was closer to 1, the analysis was reasonable; if the value was less than 0.5, the analysis was unreasonable and useless [20]. The Bartlett test of sphericity is a method to test the degree of correlation between various variables. When the significance level value is less than 0.05, the factor analysis may be useful. Therefore, analysis of the comprehensive score (0.677) via the Kaiser-Meyer-Olkin measure of sampling adequacy and Bartlett’s test of sphericity was useful and appropriate (Table 2).

#### 2.2.2. The Total Variance of the Interpretation

**Figure 2:**
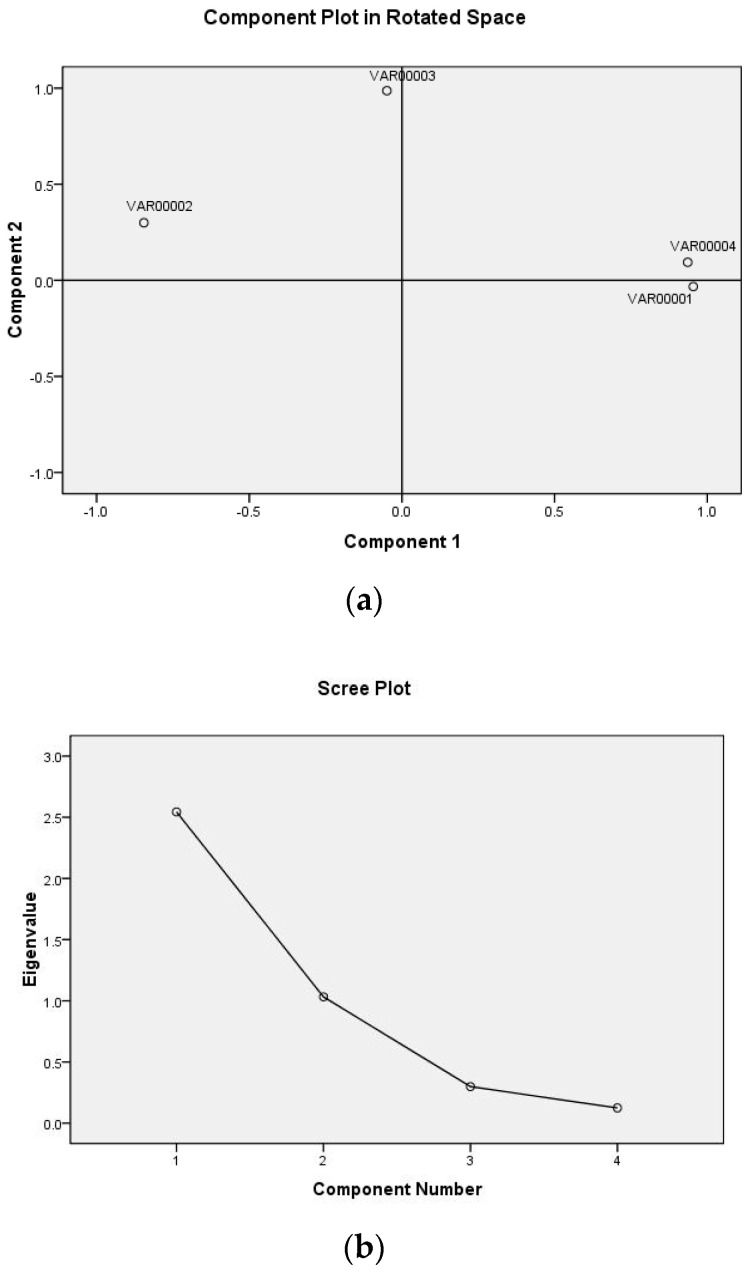
The analysis of principal component distribution (**a**) and scree plot (**b**).

The output result of the principal component distribution and scree plot are shown in Figure 2a,b. It can be seen from the load diagram that the four factors naturally clustered into two categories, V_1_, V_4,_ and V_2_ had a large load on component 1, in which V_1_ and V_4_ were positively correlated with the principal component, while V_2_ was negatively correlated with the principal component, and V_3_ had a large load on component 2. Two factors of the eigenvalue curve had an eigenvalue greater than 0. The cumulative variance contribution rate of the two factors reached 89.408% (>85%), that is, the two factors extracted reflected the information of 89.408% of the original four indexes, as shown in Table 3 [21].

**Extraction Method: Principal Component Analysis.**

#### 2.2.3. Analysis of Film Comprehensive Score

The linear relationship was obtained as follows: Z_1_ = 0.946X_1_ − 0.883X_2_ − 0.211X_3_ + 0.908X_4_, Z_2_ = 0.125X_1_ + 0.157X_2_ + 0.956X_3_ + 0.247X_4_. By analyzing the variance contribution rate, eigenvalue and eigenvector of principal components, and normalizing the data, the weights of the four indicators of the indicator film (EAB, TS, HRC and WVP) were obtained as follows: 0.31, 0.24, 0.12, and 0.33, respectively, and the comprehensive score of the indicator film performance was obtained: S = 0.31P_1_ + 0.24P_2_ + 0.12P_3_ + 0.33P_4_.

**Figure 3:**
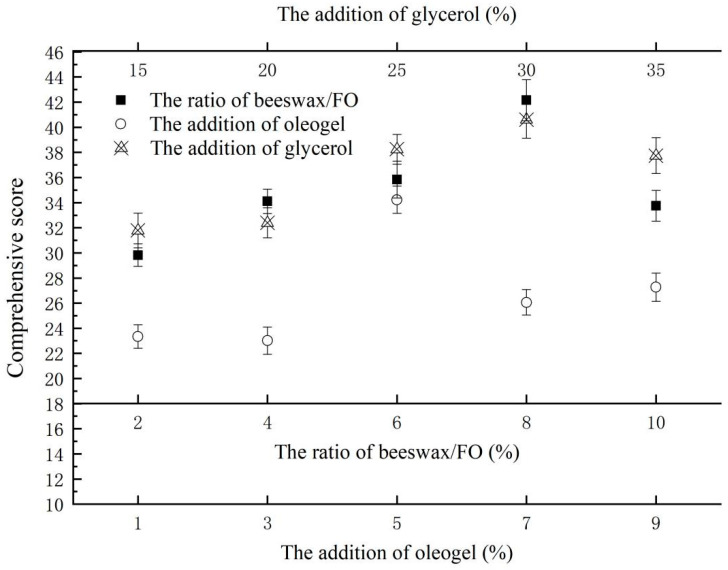
The effect of the ratio of beeswax/FO, the addition of oleogel, and the addition of glycerol on the comprehensive score of the film’s characteristics.

Via calculation, the effect of the ratio of beeswax/FO, the addition of oleogel, and the addition of glycerol on the comprehensive score of the film’s characteristics are shown in Figure 3. When the ratio of beeswax/FO was 8%, the addition of oleogel was 5%, and the addition of glycerol was 30%, all of the comprehensive scores of the film in each single factor test group had the maximum value. According to this result, the subsequent response surface optimization experiment was carried out.

### 2.3. Results of Box-Behnken Design (BBD)

**Figure 4:**
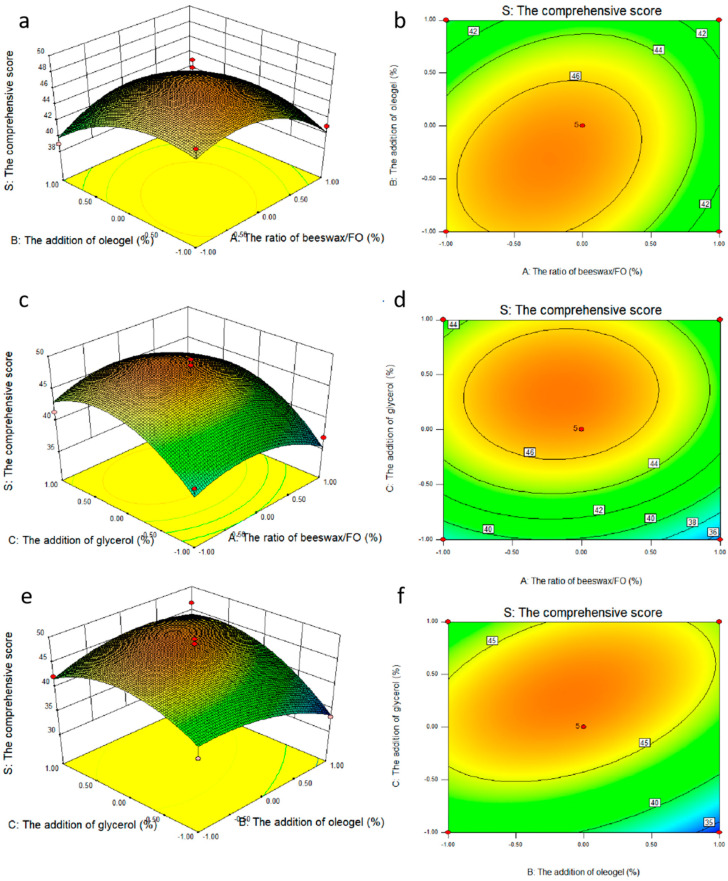
Response surface plots (**a**,**c**,**e**) and contour plots (**b**,**d**,**f**) of the effects of the interaction of various factors on the comprehensive score.

Based on single factor results, the ratio of beeswax/FO, the addition of oleogel, and the addition of glycerol were selected as the investigation factors, which were expressed as *A*, *B* and *C* respectively. The experimental design and results are shown in Table 4. The response surface plots (a,c,e) and contour plots (b,d,f) of the effects of the interaction of each factor on the comprehensive score are shown in Figure 4. The coefficients of the models were calculated, and the predicted model was as follows, respectively:

*S* = 46.98 − 1.04*A* − 1.25*B* + 2.84*C* + 1.44*AB* + 0.41*AC* + 2.46*BC* − 3.06*A*^2^ − 2.50*B*^2^ − 4.30*C*^2^ (1)

The results of ANOVA for the quadratic model are shown in Table 4 (Y), indicating that the model was highly appropriate for the prediction. The significance of each coefficient was determined via F-value and *p*-value. Generally, the larger the F value was, the smaller the *p* value was. Thus, it can be concluded from the F value of the Y model that the model was significant. The significant terms of the model characterized by the significance level also led to the lack of goodness of fit for the model. Moreover, the higher the coefficient of determination (R^2^), the better the reasonability of the model. Overall, R^2^, the fraction of the variation of the response by the model, adjusted R^2^ and regression *p*-value and lack of fit values > 0.05 all indicated that the model was well-fitted to the experimental data points [22]. The adequate precision for the system was 7.546, which was greater than 4, therefore, the adequate signal was obtained [23]. The response surface and contour maps of the pairwise interactions of factors A and B, A and C, and B and C are shown in Figure 4a, Figure 4b, Figure 4c, respectively. As can be seen from Figure 4a,b, the slope of the response surface was relatively gentle, indicating that the interaction between AB and AC was not significant (*p* > 0.05), while the slope of the response surface in Figure 4c was steeper, and the contours were distributed in a uniform and tight micro-oval shape, indicating that the interaction between BC was significant. The results of ANOVA were consistent with the result of Table 4 (P_AB_ (0.2536 > 0.05), P_AC_ (0.7352 > 0.05), and P_BC_ (0.0402 < 0.05). The interaction between factors were detected in all the samples mostly in the order BC (the addition of oleogel and the addition of glycerol) > AB (the ratio of beeswax/FO and the addition of oleogel) > AC (the ratio of beeswax/FO and the addition of glycerol).

### 2.4. Verification Test

Based on the mathematical model, the optimal experimental conditions were as follows: the ratio of beeswax/FO was 7.807%, the addition of oleogel was 4.829%, the addition of glycerol was 31.088%, and the predicted Y of the film characteristics was 47.5725. Based on the above optimal parameters, the EAB, TS, HRC, and WVP of the film were 58.21 ± 1.58%, 25.11 ± 1.24 MPa, 57.66 ± 1.44%, and (0.82 ± 0.04) ×10^−5^ g·cm/cm^2^·Kpa·h, respectively, and the actual value of the Y was 46.1624, which was close to the predicted value. The difference between the actual value and predicted value was not significant (*p* > 0.05). Therefore, the experiments showed that the model was reliable.

### 2.5. Analysis of Scanning Election Microscopy (SEM)

**Figure 5:**
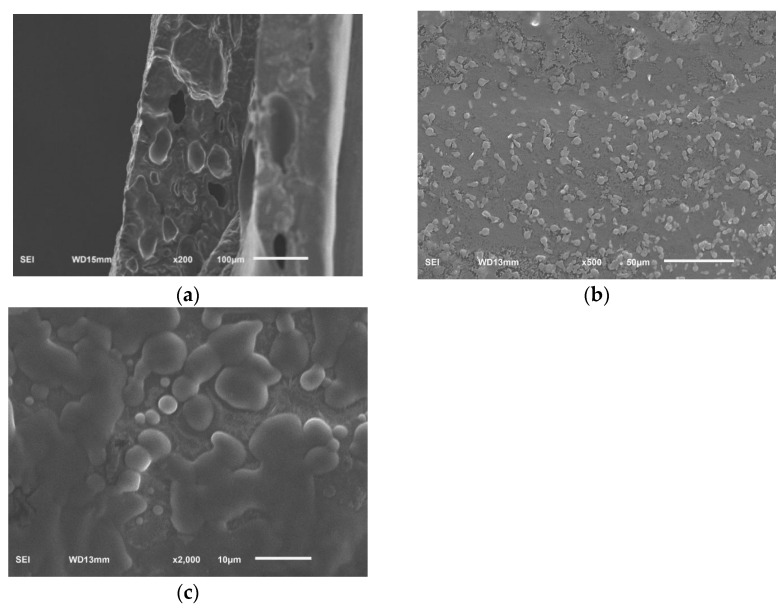
SEM, surface (viewed at magnification 500×) (**a**), cross section (viewed at magnification 200×) (**b**), cross section (viewed at magnification 2000×) (**c**), images of sodium alginate films added with linseed oil/beeswax oleogel.

The surface morphology and cross section morphology of sodium alginate film with linseed oil/beeswax oleogel prepared under optimal experimental conditions were characterized via SEM (Figure 5). The result showed that the superficial structure of the film was uneven, grainy, and rough (Figure 5a). The film had a relatively uniform, continuous, and dense structure, and had tiny pores (Figure 5b,c). This may be due to the hydrophobicity of oleogels, which accumulate into hydrophobic clumps in the original hydrocolloid matrix, resulting in uneven mixing of the water phase and the oil phase [24]. A heterogeneous surface and rough cross appearance were also reported by Yousuf et al. [3] for karaya gum-based films added with schisandra chinensis oil and its oleogel.

### 2.6. Physical and Chemical Quality Changes of Decapterus Maruadsi during Storage

#### 2.6.1. Change of Drip Loss

**Figure 6:**
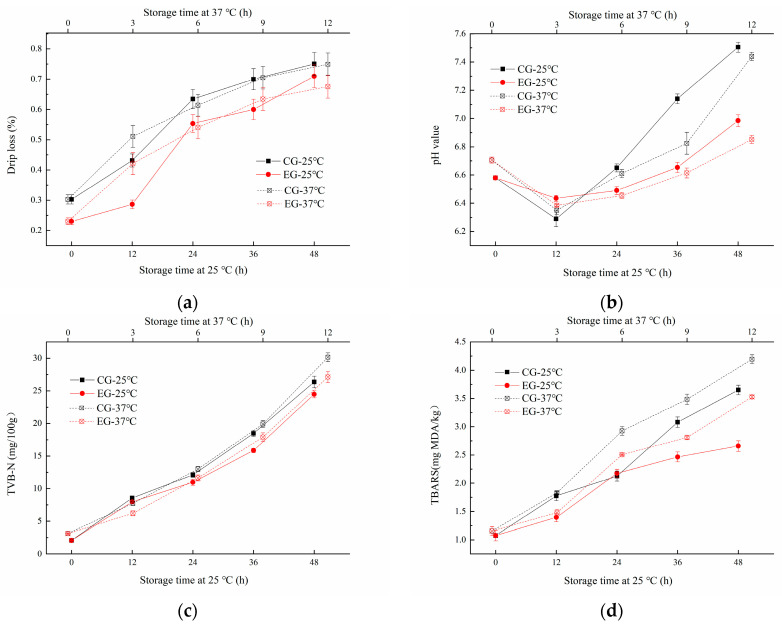
Change of drip loss (**a**), pH (**b**), TVB-N (**c**), and TBARS (**d**) of *Decapterus maruadsi* during storage at different temperatures.

The *Decapterus maruadsi* was packaged with two types of films, which was the sodium alginate film added with linseed oil/beeswax oleogel as the experimental group (EG), and the other was sodium alginate film only with added linseed oil as a control group (CG). It can be seen from Figure 6a that the drip loss of EG and CG both increased with the extension of storage time at 25 °C and 37 °C (*p* < 0.05). The dripping loss of CG (0.30~0.75%) was always higher than that of EG (0.23~0.71%) during the 0~48 h at 25 °C storage. The changes at 37 °C were similar to those at 25 °C. This suggested that the addition of linseed oil/beeswax oleogel can significantly reduce the drip loss of the samples. This effect was superior to the addition of linseed oil alone, which may be related to the hydrophobic behavior of the added oil/oleogel [16].

#### 2.6.2. Change of pH Value

The changes of pH of the samples during storage were shown in Figure 6b. The pH of EG and CG decreased first in the prestorage period, then increased significantly during the late storage period. When at 25 °C storage temperature, the pH value of CG varied from 6.29 to 7.14, and that of EG varied from 6.45 to 6.99. This phenomenon may be speculated that in the early stage of storage, bacteria used small molecules of organic matter to ferment fish and produce acid. With the progress of storage, proteins in fish will be further decomposed into a nitrogen-containing alkaline with small molecules under the action of bacteria [25]. Similarly, the pH value of CG varied from 6.35 to 7.44, and that of EG varied from 6.39 to 6.99, indicating that the pH value of EG changed more steadily than that of CG, and the sodium alginate film added with linseed oil/beeswax oleogel can inhibit the degeneration of *Decapterus maruadsi* during storage more effectively.

#### 2.6.3. Change of Total Volatile Basic Nitrogen (TVB-N)

TVB-N content is an important index to judge the freshness of meat. If the value of sample is greater than 25 mg/100 g, it is considered to be rotten meat [26]. The change of TVB-N of EG and CG under different storage conditions are shown in Figure 6c. The TVB-N value of all tested samples increased with the extension of storage at 25 °C and 37 °C (*p* < 0.05). After 48 h storage at 25 °C, the TVB-N value of CG increased to 26.35 mg/100 g, while that of EG increased to 24.48 mg/100 g. During 12 h storage at 36 °C, the TVB-N value of CG increased to 30.16 mg/100 g, while that of EG increased to 27.12 mg/100 g. Under the same storage conditions, the TVB-N value of CG increased faster and higher than EG, which indicated that the addition of linseed oil/beeswax oleogel can effectively inhibit the deterioration of proteins of *Decapterus maruadsi*.

#### 2.6.4. Change of Thiobarbituric Acid Reactive Substance (TBARS)

As shown in Figure 6d, the TBARS of EG and CG increased significantly with the extension of storage (*p* < 0.05). The two-group increased significantly during the period of 24 h to 36 h at 25 °C, as well as the storage of 2 h to 4 h at 37 °C. CG changed more rapidly than EG during storage. Similar results were reported by Hamdani, et al. [27] and Yousuf [3]; the addition of the oleogel to the edible film can make the film have a more significant antioxidant effect, and the effect of addition in the form of oleogel or only pure oil was quite different. The stronger the antioxidant properties were, the slower the deterioration of fats and proteins. Thus, the novel oleogel added to the film can enhance the preservation effect of the film on fish products, improve the quality of *Decapterus maruadsi* during storage, and extend the shelf-life.

#### 2.6.5. Change of Fatty Acids Composition

A total of 22 kinds of fatty acids of *Decapterus maruadsi* were isolated and identified via GC, and the main PUFA contained linoleic acid (C18:2n−6), eicosenoic acid (C20:3n−6), eicosapentaenoic acid (EPA, C20:5n−3), and docosahexaenoic acid (DHA, C22:6n−3). The main SFA contained palmitic acid (C16:0), stearic acid (C18:0), and docosanoic acid (C22:0), etc. (Table 5). During storage at 25 °C, PUFA content of EG decreased from 51.06% to 41.39%, CG decreased from 51.06% to 40.2%. At 37 °C, PUFA content of EG decreased from 51.06% to 39.37% and CG decreased from 51.06% to 36.41%. The above changes were due to the decrease of the change of main PUFA (C18:2n−6, C20:3n−6, C20:5n−3, and C22:6n−3) and the increase of SFA (C16:0, C18:0, C22:0, etc.). The C18:1n−9 of all the samples had an overall increasing trend, but the total amount of MUFA did not change much, and the changing trend was not obvious. The variation range of fatty acid composition in EG was smaller than that of CG, which suggested that the sodium alginate film added with linseed oil/beeswax oleogel can effectively prevent the oxidation of fatty acids of *Decapterus maruadsi* during storage; it was consistent with the above results of TBARS.

#### 2.6.6. Correlation Analysis of the Change of Each Index

**Figure 7:**
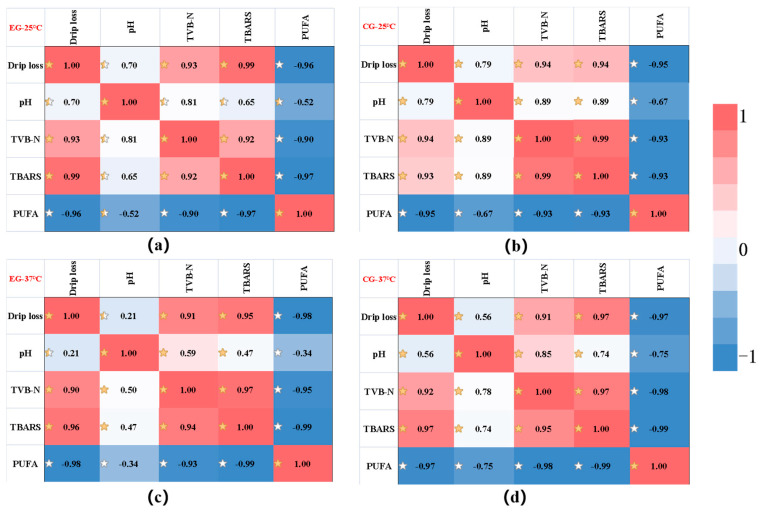
The correlation analysis of the change of each index. ((**a**): the result of EG at 25 °C; (**b**): the result of CG at 25 °C; (**c**): the result of EG at 37 °C; (**d**): the result of EG at 37 °C. The correlations were represented by different stars shadows).

In order to screen suitable indicators for product shelf-life prediction, the relationship between various indicators of *Decapterus maruadsi* preserved via the sodium alginate films were analyzed, and the correlation analysis between various indicators under different conditions was established. As shown in the Figure 7, the Pearson correlation coefficient (r) varied between −1 and 1, and the degree of correlation was better explained using the color scale. In the figure, TVB-N was positively correlated with drip loss, pH and TBARS, while it negatively correlated with the effect of PUFA. In addition, the correlation between TVB-N and various indicators was high, and most of them were greater than 0.9. In view of this, TVB-N was selected to construct a shelf-life prediction model for *Decapterus maruadsi* preserved via the sodium alginate films.

### 2.7. Establishment and Verification of the Kinetic Model of Quality Change in Decapterus Maruadsi Preserved via the Sodium Alginate Films

#### 2.7.1. Regression Equation of TVB-N of Decapterus Maruadsi Preserved via the Sodium Alginate Films under Different Storage Conditions

The fitting linear regression equation, regression coefficient R^2,^ and rate constant k values can be seen from Table 6. The multiple correlation coefficient R^2^ of the regression equations established at storage temperatures of 25 °C (298.15 K) and 37 °C (310.15 K) were both greater than 0.90, indicating that the regression equation had a high degree of fit. At the same time, the obtained rate constant *k* values were 0.43903 (EG at 25 °C), 1.98921 (EG at 37 °C), 0.48708 (CG at 25 °C), and 2.20737 (CG at 25 °C), respectively.

The linear equation of EG y = −11.64310015x + 38.22796 was obtained via plotting the inverse 1/T of the storage temperature with lnK. The activation energy Ea of the peroxide value was 9.6805 × 10^4^ J/mol and the prefactor k_0_ was 4.00122 × 10^16^. On this basis, the Arrhenius equation of EG, the kinetic equation and the shelf-life prediction equation between the peroxidation rate constant k and the storage temperature T during the storage of *Decapterus maruadsi* preserved via the sodium alginate films under different storage conditions were established as follows:

Arrhenius equation:

(2) $$A=Ao\times e^{(\frac{-9.6805\times 10^{4}}{8.3144T})t}$$

First-order dynamic equation:

(3) $$k=4.00122\times 10^{16}\times e^{\frac{-9.6805\times 10^{4}}{8.3144T}}$$

Shelf-life prediction formula:

(4) $$SL=\frac{\ln\left(TVB-N\right)-ln(TVB-No)}{4.00122\times 10^{16}\times exp(\frac{-9.6805\times 10^{4}}{8.3144T})}$$

Similarly, the linear equation of CG y = −11.64466872x + 38.33708 was obtained via plotting the inverse 1/T of the storage temperature with lnK. The activation energy Ea of the peroxide value was 9.6818 × 10^4^ J/mol and the prefactor k_0_ was 4.46254 × 10^16^. On this basis, the Arrhenius equation of CG, the kinetic equation and the shelf-life prediction equation between the peroxidation rate constant k and the storage temperature T during the storage of *Decapterus maruadsi* preserved via the sodium alginate films under different storage conditions were established as follows:

Arrhenius equation:

(5) $$A=Ao\times e^{(\frac{-9.6818\times 10^{4}}{8.3144T})t}$$

First-order dynamic equation:

(6) $$k=4.46254\times 10^{16}\times e^{\frac{-9.6818\times 10^{4}}{8.3144T}}$$

Shelf-life prediction formula:

(7) $$SL=\frac{\ln\left(TVB-N\right)-ln(TVB-No)}{4.46254\times 10^{16}\times exp(\frac{-9.6818\times 10^{4}}{8.3144T})}$$

According to the shelf-life prediction equation, when the storage temperature, initial *TVB*-*N* value, and final *TVB*-*N* value were determined, the storage time of *Decapterus maruadsi* preserved via the sodium alginate films under different storage conditions under a certain temperature condition can be calculated, so that its shelf-life can be predicted. In addition, the storage temperature, initial *TVB*-*N* value, and storage time of *Decapterus maruadsi* preserved via the sodium alginate films under different storage conditions can also be used to calculate the final TVB-N value after a certain period of storage at the determined storage temperature, so as to monitor the change of its quality.

#### 2.7.2. Prediction of Kinetic Model of TVB-N value of Decapterus Maruadsi Preserved via the Sodium Alginate Films under Different Storage Conditions

According to the national limited standard (the TVB-N value was 25 mg/100 g), the predicted shelf-life of EG at 25 °C and 37 °C was 56.6365 (approx. 56.6 h) and 12.5000 d (approx. 12.5 h). The predicted shelf-life of CG at 25 °C and 37 °C was 42.7086 (approx. 42.7 h) and 9.4241 h (approx. 9.4 h), which may provide the theoretical references for the safe storage of products. On the whole, the *Decapterus maruadsi* preserved via the sodium alginate films added with linseed oil/beeswax oleogel had good storage resistance, and the *Decapterus* preserved with sodium alginate film added with linseed oil/beeswax oleogel had a longer shelf-life than that preserved with sodium alginate film added only with linseed oil (Table 6).

## 3. Conclusions

The novel oleogel can be successfully applied to the edible film, which can improve the barrier property and texture characteristics of the film. When the ratio of beeswax/FO was 7.807%, the addition of oleogel was 4.829%, and the addition of glycerol was 31.088%, the comprehensive score of the film characteristics had the maximum, and the EAB, TS, HRC, and WVP of the film were 58.21 ± 1.58%, 25.11 ± 1.24 MPa, 57.66 ± 1.44%, and (0.82 ± 0.04) × 10^−5^ g·cm/cm^2^·KPa·h, respectively. In comparison to control, the sodium alginate films incorporated with linseed oil/beeswax oleogel can effectively inhibit the quality deterioration of *Decapterus maruadsi*, which had a longer shelf-life. The preservation effect of the film incorporated with linseed oil/beeswax oleogel was better than that of film incorporated only with linseed oil. In a word, linseed oil/beeswax oleogel can be used to develop novel types of biodegradable films, which can reduce water vapor permeability, keep excellent mechanical properties, and effectively extend the shelf-life of *Decapterus maruadsi*.

## 4. Materials and Methods

### 4.1. Materials

FO was purchased from Lu Hua Co. Ltd. (Yantai, China). Sodium alginate (food grade) was purchased from Zhejiang Yi Nuo Biotechnology Co., Ltd. Hangzhou China. Beeswax (yellow) was brought from Cangzhou bee wing wax industry Co., Ltd. (Cangzhou, China). Water was purified with a Milli-Q water purification system (Millipore Co., Ltd., Burlington, MA, USA). Boron trifluoride methanol solution and 37 fatty acid methyl ester mixed standard were brought from Shanghai Anpu Experimental Technology Co., Ltd., (Shanghai, China). Other chemicals used were of analytical grade, and brought from Xilong Scientific Co., Ltd. (Shantou, China). Fresh *Decapterus maruadsi* (about 1.0 kg weight) were purchased from RT-Mart (Zhangzhou, Fujian, China), and transported to the laboratory alive.

### 4.2. Oleogel Preparation

Oleogel was prepared with yellow beeswax as the oleogelator at a concentration of 2~10%, corresponding to the FO. The beeswax was accurately weighed and dispersed in FO. Then, heat the mixture at 80 °C in a water bath (DF-101S, Shanghai Lichen Bangxi Instrument Technology Co., Ltd., Shanghai, China), and stir gently for 20 min to blend well. Then, cool the mixture to room temperature to get oleogel [3].

### 4.3. Oleogel Preparation

Based on the preliminary experiments, 1.5% sodium alginate was considered to be the appropriate concentration for preparing films. Accurately weigh the sodium alginate, and dissolve in distilled water; the mixture was kept in a hot water bath for 30 min at 80 °C. Glycerol was added to the mixture as a plasticizer at a concentration of 15~35% of the weight of film. Add the oleogel at different levels (1~9%), and stir the mixture at 1000 rpm for 20 min at 60 °C on a magnetic stirrer (DF-101S, Shanghai Lichen Bangxi Instrument Technology Co., Ltd., Shanghai, China). The mixture was cooled and treated via ultrasound for 20 min to remove air bubbles (KQ3200E, Xinzhi biotechnology Co., Ltd., Ningbo, China). Take 25 g of the final film liquid mixture and pour it onto a plate with a diameter of 90 mm and dry it in a hot air stove at 30 °C for 24 h. Before analysis, the film was stripped from the plate and stored in a dryer containing saturated sodium bromide solution for at least 48 h.

### 4.4. Single Factor Experiment

Investigate the effects of three single factors (the ratio of beeswax/FO (2~10%), the addition of oleogel (1~9%), and the addition of glycerol (15~35%) on the EAB, TS, HRC, and WVP of the film.

### 4.5. Characterization of Films

#### 4.5.1. Thickness

The thickness of films was tested with a digital micrometer (BMD-25D, Mitutoyo Mfg. Co., Ltd., Kawasaki, Japan). Five random regions were selected and the average values was calculated.

#### 4.5.2. Mechanical Properties

The TS and EAB were determined via the texture analyzer (CT3-10K, American Bollerfly Company, Middleboro, MA, USA). The films were cut into strips of 8.0 cm × 2.0 cm. The initial clamping distance was 60 mm and the crosshead velocity was 50 mm/min [28].

#### 4.5.3. HRC

Take an appropriate amount of edible film and dissolve it with water to prepare the 0.8% film solution. The 50 μL sample solution was accurately removed, and 50 μL FeSO_4_ (6 mmol/L) and 100 μL H_2_O_2_ (6 mmol/L) were added, then fully shaken and placed for 10 min. Add 50 μL salicylic acid (6 mmol/L), shake with partial vibration, and place at room temperature for 30 min. The absorbance was tested at 510 nm. The HRC was calculated according to Formula (8):

(8) $$HRC=\frac{A_{0}-A_{i}}{A_{0}}$$

where A_0_ was the light absorption value of the blank tube, A_i_ was the light absorption value of the sample tube.

#### 4.5.4. WVP

WVP was carried out according to Fasihi, Fazilati, Hashemi, and Noshirvani [29]. Briefly, add 20 g silica gel to the centrifuge tube to keep the RH value of the tube at 0%, then the centrifugal tube was sealed with a thin film with a diameter slightly larger than the centrifugal tube. The centrifugal tube was placed in an environment with a temperature of 25 °C and an RH value of 60%, and the weight change of the weighing cup was recorded every 2 h. The WVP values were calculated as Formula (9):

(9) $$WVP=\frac{1}{A}(\frac{∆W}{t})\times \frac{T}{P({RH}_{1}-{RH}_{2})}$$

where ΔW represented the weight change before and after measurement (g); t was the time (s); A was the test area (m^2^); P was the saturation vapor pressure of water (3.169 × 103 Pa at 25 °C); RH_1_ denoted the relative humidity value in the desiccators and RH_2_ denoted the relative humidity value in the permeation cell; and T was the film thickness (mm).

#### 4.5.5. Calculation of Comprehensive Score of Edible Film Characteristic

Referring to the method of Jiang et al. [30], the SPSS V23.0 software was used to randomly select 9 groups of data from the single-factor experiment. The comprehensive score of film characteristic was indicated by the membership degree of the comprehensive score and the analysis of the principal component. The principal component analysis was mainly used to determine the weight of each index.

Membership degree was calculated according to Formulas (10) and (11):

(10) $$P=\frac{A_{i}-A_{min}}{A_{max}-A_{min}}$$

(11) $$P=1-\frac{A_{i}-A_{min}}{A_{max}-A_{min}}$$

where P was membership degree; A_i_ was the corresponding index value; A_max_ was the maximum value of the corresponding indicator; and A_min_ was the minimum value of the corresponding indicator.

The values of the positive effect (TS, EAB, and HRC) were calculated using Equation (10). The values of the negative effect (WVP) were calculated via Equation (11).

The comprehensive score of edible film characteristic (S) was calculated according to Formula (12):

(12) $$S=a_{1}P_{1}+a_{2}P_{2}+a_{3}P_{3}+a_{4}P_{4}$$

In the formula, P_1_ to P_4_ were the membership values of TS, EAB, HRC, and WVP, respectively; a1 to a4 were the weights of the four indicators.

#### 4.5.6. SEM

Thin films were fractured in liquid nitrogen to prepare the cross section. After gold spraying under vacuum, the surface and cross section morphology of films were observed via SEM (Hitachi SU-8000, Tokyo, Japan) at an accelerating voltage of 20 kV.

### 4.6. BBD

The response values were the comprehensive score of the film characteristics (S), and the three variables were the ratio of beeswax/FO (7–9%), the addition of oleogel (4–6%), and the addition of glycerol (26–34%).

### 4.7. The Application of the Film on Preservation of Decapterus maruadsi

#### 4.7.1. Storage Experiment

Each fresh *Decapterus maruadsis* was humanely slaughtered with ice-water slurry (2.4 kg ice: 3.6 L water: 1 kg fish) for 20 min, and immediately beheaded, scaled, eviscerated, and washed with tap water. The fish was collected and stored in ice for 24 h for further treatment. Before experimentation, the fish was cut into 2 cm × 2 cm × 0.2 cm fillets. The methodological protocol of the current study was approved by the welfare and ethics committee in animal experiment of College of Biological Science and Technology, Minnan Normal University.

The samples were stored at 25 °C for 0, 12, 24, 36, and 48 h, and at 37 °C for 0, 2, 4, 6, and 8 h, respectively. The changes of water content, TVB-N value, pH value, TBARS value, and fatty acid composition were determined at each time period. The *Decapterus maruadsi* preserved with film added with oleogel was the experimental group (EG), and the fish preserved with film added only with FO was the control group (CG).

#### 4.7.2. Change of Drip Loss

The change of drip loss was estimated as a percentage of the original weight shown in Equation (13):

(13) $$Driploss\left(\%\right)=\frac{initial\;weight-final\;weight}{initial\;weight}\times 100$$

#### 4.7.3. TVB-N Value

The content of TVB-N was determined via the Kjeldahl method (Kjeltec 8400, FOSS, Hiller) [31]. The result was expressed as mg of nitrogen per 100 g of sample.

#### 4.7.4. pH Value

The calibrated portable pH meter (Testo735-2, Testo AG, Baden-Wurttemberg, Germany) was used to test the pH value. Each sample was measured at three different locations and the results were expressed as the average of the three trials [32].

#### 4.7.5. TBARS Analysis

Add 45 mL of frozen 20% (*w*/*v*) trichloroacetic acid (TCA) to 5.0 g *Decapterus maruadsi* samples, extract at room temperature for about 1 h, then centrifuge at 4000× g for 15 min, add 5 mL of supernatant to 5 mL of 0.02 M TBA, heat at 95 °C for 30 min, and cool to room temperature with tap water. TBARS value was expressed as mg of malondialdehyde (MDA) per kg of sample.

#### 4.7.6. Fatty Acid Composition

The fatty acid extraction, methyl ester reaction and gas chromatographic determination were referred to as in Xue [33].

### 4.8. Establishment of Kinetic Model of Decapterus Maruadsi during Storage

#### 4.8.1. First-Order Kinetic Equation

Most of the changes in food processing follow the zero-order or first-order reaction model [34]. The first-order reaction kinetics equation is shown in Equation (14):

A = A_0_ekt (14)

A: the quality index values at t d;

A_0_: the quality index values at 0 d;

K: the change rate constant of storage quality index;

T: the storage time of the sample (d).

#### 4.8.2. Arrhenius Equation

The Arrhenius equation can reflect the relationship between the change rate constant (k) and the thermodynamic temperature (*T*). When the rate constant under different temperature conditions is calculated, a line with a slope of −*Ea*/*R* and a linear equation with y-intercept of lnk_0_ can be fitted via drawing lnk for 1/*T*, and the reaction activation energy Ea and the former factor k_0_ can be calculated. The Arrhenius equation is shown in Equation (15):

(15) $$k=ko\times exp(-\frac{Ea}{RT})$$

After taking the logarithm of Formula (16):

(16) $$lnk=lnko-\frac{Ea}{RT}$$

K_0_: the former factor (frequency factor) of the equation;

*Ea*: the activation energy/(J/moL) of the reaction of storage quality index change;

*T*: the absolute temperature/K;

*R*: the gas constant (8.314, 4 J/(mol•K));

Both k_0_ and *Ea* are empirical constants related to the physical nature of the reaction system.

By combining the first-order kinetic equation and Arrhenius equation, the shelf-life of products can be predicted theoretically.

### 4.9. Statistical Analysis

The data collected were subjected to ANOVA using Excel^®^ 2010 software and Tukey tests (with a 95% confidence interval) to evaluate differences between the results. The principal component analysis used SPSS 17.0. The optimization experiment was conducted via design-expert 8.0.6.

## Figures and Tables

**Table 1:** Test data of contribution rate via principal component analysis.

No.	EAB (%)	TS (MPa)	HRC(%)	WVP (10^−5^ g·cm/cm^2^·KPa·h)
1	31.7 ± 0.74	25.68 ± 1.53	56.28 ± 0.36	1.07 ± 0.06
2	45 ± 1.66	12.42 ± 1.58	57.58 ± 0.7	1.17 ± 0.05
3	58.03 ± 2.27	11.55 ± 1.22	60.42 ± 0.87	1.48 ± 0.04
4	21.87 ± 1.22	19.2 ± 0.94	32.98 ± 0.97	0.77 ± 0.05
5	43.4 ± 1.33	13.46 ± 0.76	50.77 ± 1.01	1.13 ± 0.06
6	28.78 ± 0.75	20.78 ± 1.04	29.97 ± 1.56	0.53 ± 0.07
7	42.3 ± 1.13	10.51 ± 1.05	48.72 ± 1.55	1.27 ± 0.07
8	57.3 ± 1.16	9.35 ± 0.89	45.33 ± 1.69	1.31 ± 0.08
9	54.93 ± 1.47	15.35 ± 1.18	52.86 ± 1.99	1.28 ± 0.06

**Table 2:** KMO and Bartlett spherical test for the dimension reduction model of edible film characteristics index.

Kaiser-Meyer-Olkin Measure of Sampling Adequacy.		0.677
Bartlett’s Test of Sphericity	Approx. Chi-Square	13.558
	df	6
	Sig.	0.035

**Table 3:** Eigenvalues and cumulative variance contribution rates of the related components.

Component	Initial Eigenvalues			Extraction Sums of Squared Loadings		
	Total	% of Variance	Cumulative %	Total	% of Variance	Cumulative %
1	2.544	63.594	63.594	2.544	63.594	63.594
2	1.033	25.815	89.408	1.033	25.815	89.408
3	0.299	7.478	96.886			
4	0.125	3.114	100			

**Table 4:** Experimental design plan, results, and variance analysis of response surface experiment.

Test	The Ratio of Beeswax/FO (A)	The Addition of Oleogel (B)	The Addition of Glycerol (C)	Comprehensive Score (S)
1	1 (9)	0 (5)	1 (34)	39.445
2	0 (8)	−1 (4)	−1 (26)	46.599
3	−1 (7)	1 (6)	0 (30)	49.378
4	−1	0	1	48.447
5	0	0	0	46.361
6	0	0	0	44.954
7	0	1	1	38.682
8	0	1	−1	33.187
9	1	−1	0	40.971
10	−1	−1	0	46.291
11	0	0	0	39.004
12	0	0	0	41.517
13	1	0	−1	36.911
14	0	−1	1	42.245
15	1	1	0	39.424
16	0	0	0	45.752
17	−1	0	−1	40.607
Source	Sum of squares	Mean square	F Value	*p* Value
Model	278.17	30.91	5.81	0.0151
A	8.7	8.7	1.63	0.2418
B	12.44	12.44	2.34	0.1702
C	64.66	64.66	12.15	0.0102
AB	8.24	8.24	1.55	0.2536
AC	0.66	0.66	0.12	0.7352
BC	24.25	24.25	4.56	0.0402
A^2^	39.35	39.35	7.39	0.0298
B^2^	26.29	26.29	4.94	0.0617
C^2^	77.9	77.9	14.63	0.0065
Residual	37.26	5.32		
Lack of Fit	23.36	7.79	2.24	0.2257
Pure Error	13.9	3.47		
Cor Total	315.43			

**Table 5:** Change of fatty acids composition of *Decapterus maruadsi* during storage at 25 °C and 37 °C.

Fatty Acids	EG-25 °C					CG-25 °C					EG-37 °C					CG-37 °C				
	0 h	12 h	24 h	36 h	48 h	0 h	12 h	24 h	36 h	48 h	0 h	12 h	24 h	36 h	48 h	0 h	12 h	24 h	36 h	48 h
C12:0	0.77 ± 0.09 ^c^	0.85 ± 0.09 ^b^	1.00 ± 0.01 ^a^	0.87 ± 0.08 ^b^	0.81 ± 0.04 ^b^	0.77 ± 0.09 ^c^	1.26 ± 0.03 ^a^	1.11 ± 0.02 ^b^	1.12 ± 0.02 ^b^	0.6 ± 0.04 ^d^	0.77 ± 0.09 ^c^	0.81 ± 0.03 ^c^	0.62 ± 0.09 ^d^	0.97 ± 0.06 ^b^	1.16 ± 0.05 ^a^	0.77 ± 0.09 ^b^	0.71 ± 0.01 ^b^	0.56 ± 0.09 ^c^	0.96 ± 0.08 ^a^	0.99 ± 0.03 ^a^
C14:1n−9	0.94 ± 0.05 ^a^	0.44 ± 0.02 ^b^	0.33 ± 0.02 ^c^	0.35 ± 0.01 ^c^	0.33 ± 0.03 ^c^	0.94 ± 0.05 ^b^	1.38 ± 0.1 ^a^	0.78 ± 0.1 ^c^	0.57 ± 0.06 ^d^	0.28 ± 0.09 ^e^	0.94 ± 0.05 ^b^	0.77 ± 0.09 ^c^	0.61 ± 0.02 ^d^	0.55 ± 0.09 ^e^	1.02 ± 0.01 ^a^	0.94 ± 0.05 ^a^	0.75 ± 0.1 ^c^	0.56 ± 0.07 ^d^	0.82 ± 0.01 ^b^	0.56 ± 0.1 ^d^
C15:0	0.41 ± 0.09 ^c^	0.82 ± 0.01 ^b^	0.93 ± 0.09 ^a^	0.85 ± 0.01 ^b^	0.83 ± 0.02 ^b^	0.41 ± 0.09 ^c^	0.93 ± 0.07 ^b^	0.92 ± 0.04 ^b^	0.9 ± 0.07 ^b^	1.23 ± 0.04 ^a^	0.41 ± 0.09 ^c^	0.69 ± 0.1 ^b^	0.72 ± 0.04 ^b^	0.81 ± 0.04 ^a^	0.89 ± 0.07 ^a^	0.41 ± 0.09 ^d^	0.64 ± 0.08 ^b^	0.65 ± 0.03 ^b^	0.54 ± 0.04 ^c^	0.73 ± 0.04 ^a^
C15:1	3.3 ± 0.07 ^b^	3.26 ± 0.04 ^b^	3.46 ± 0.01 ^a^	2.83 ± 0.04 ^c^	2.8 ± 0.01 ^c^	3.3 ± 0.07 ^b^	3.53 ± 0.06 ^a^	3.09 ± 0.03 ^c^	3.42 ± 0.08 ^a^	0.28 ± 0.04 ^d^	3.3 ± 0.07 ^a^	3.29 ± 0.1 ^a^	2.65 ± 0.05 ^b^	2.12 ± 0.06 ^c^	1.65 ± 0.05 ^d^	3.3 ± 0.07 ^b^	3.06 ± 0.01 ^c^	3.82 ± 0.03 ^a^	3.34 ± 0.02 ^b^	2.13 ± 0.02 ^d^
C16:0	18.53 ± 0.17 ^e^	23.53 ± 0.13 ^d^	24.29 ± 0.17 ^c^	25.64 ± 0.13 ^b^	27.32 ± 0.13 ^a^	18.53 ± 0.17 ^d^	20.54 ± 0.17 ^c^	23.39 ± 0.18 ^b^	25.91 ± 0.19 ^b^	27.08 ± 0.12 ^a^	18.53 ± 0.17 ^d^	19.28 ± 0.02 ^c^	23.96 ± 0.08 ^b^	25.33 ± 0.09 ^a^	26.25 ± 0.05 ^a^	18.53 ± 0.17 ^e^	19.05 ± 0.13 ^d^	23.17 ± 0.17 ^c^	25.69 ± 0.16 ^b^	29.06 ± 0.13 ^a^
C16:1	4.06 ± 0.06 ^d^	3.77 ± 0.04 ^e^	4.41 ± 0.02 ^c^	5.5 ± 0.04 ^a^	5.15 ± 0.02 ^b^	4.06 ± 0.06 ^b^	3.36 ± 0.09 ^d^	3.81 ± 0.06 ^c^	4.73 ± 0.08 ^a^	4.86 ± 0.06 ^a^	4.06 ± 0.06 ^a^	3.53 ± 0.01 ^c^	2.96 ± 0.07 ^d^	3.22 ± 0.02 ^b^	2.89 ± 0.05 ^e^	4.06 ± 0.06 ^b^	4.66 ± 0.09 ^a^	3.05 ± 0.08 ^d^	3.69 ± 0.02 ^c^	2.82 ± 0.02 ^e^
C18:0	7.66 ± 0.08 ^a^	6.1 ± 0.05 ^d^	7.07 ± 0.04 ^b^	6.26 ± 0.06 ^c^	6.91 ± 0.09 ^b^	7.66 ± 0.08 ^c^	9.15 ± 0.08 ^a^	7.45 ± 0.09 ^d^	7.29 ± 0.03 ^e^	8.2 ± 0.08 ^b^	7.66 ± 0.08 ^b^	7.68 ± 0.06 ^b^	7.96 ± 0.11 ^a^	7.78 ± 0.07 ^b^	7.4 ± 0.06 ^c^	7.66 ± 0.08 ^b^	8.47 ± 0.01 ^a^	6.88 ± 0.08 ^d^	7.17 ± 0.05 ^c^	6.7 ± 0.08 ^e^
C18:1n−9	10.05 ± 0.09 ^d^	9.8 ± 0.03 ^e^	11.11 ± 0.13 ^c^	12.08 ± 0.11 ^b^	12.35 ± 0.11 ^a^	10.05 ± 0.09 ^d^	10.124 ± 0.09 ^d^	11.16 ± 0.02 ^c^	11.66 ± 0.06 ^a^	11.32 ± 0.13 ^b^	10.05 ± 0.09 ^e^	11.31 ± 0.08 ^d^	12.26 ± 0.10 ^c^	13.26 ± 0.12 ^b^	15.19 ± 0.21 ^a^	10.05 ± 0.09 ^e^	11.82 ± 0.18 ^d^	12.72 ± 0.14 ^c^	14.74 ± 0.18 ^b^	17.15 ± 0.18 ^a^
C18:2n−6	18.83 ± 0.11 ^a^	16.74 ± 0.11 ^b^	15.94 ± 0.12 ^c^	15.26 ± 0.11 ^d^	15.33 ± 0.14 ^d^	18.83 ± 0.11 ^a^	16.87 ± 0.18 ^b^	14.34 ± 0.19 ^d^	14.11 ± 0.11 ^d^	15.12 ± 0.13 ^c^	18.83 ± 0.11 ^a^	18.12 ± 0.16 ^b^	16.89 ± 0.14 ^c^	16.23 ± 0.24 ^d^	15.98 ± 0.25 ^e^	18.83 ± 0.11 ^a^	16.99 ± 0.12 ^b^	16.12 ± 0.19 ^c^	15.88 ± 0.18 ^d^	15.46 ± 0.19 ^e^
C18:3n−6	10.84 ± 0.15 ^a^	10.7 ± 0.11 ^a^	10.39 ± 0.18 ^b^	9.75 ± 0.04 ^c^	9.87 ± 0.08 ^c^	10.84 ± 0.15 ^a^	9.88 ± 0.08 ^b^	7.91 ± 0.11 ^c^	6.88 ± 0.17 ^e^	7.32 ± 0.27 ^d^	10.84 ± 0.15 ^a^	10.3 ± 0.1 ^b^	9.87 ± 0.03 ^c^	9.83 ± 0.06 ^c^	9.24 ± 0.12 ^d^	10.84 ± 0.15 ^a^	10.68 ± 0.15 ^b^	10.56 ± 0.14 ^c^	10.06 ± 0.14 ^d^	8.61 ± 0.19 ^e^
C18:3n−3	3.93 ± 0.04 ^a^	2.59 ± 0.05 ^b^	1.85 ± 0.05 ^c^	1.49 ± 0.07 ^d^	1.26 ± 0.07 ^e^	3.93 ± 0.04 ^a^	2.33 ± 0.01 ^b^	2.16 ± 0.02 ^c^	2.1 ± 0.01 ^c^	1.96 ± 0.05 ^c^	3.93 ± 0.04 ^a^	3.78 ± 0.09 ^b^	3.15 ± 0.06 ^d^	3.31 ± 0.04 ^c^	3.35 ± 0.06 ^c^	3.93 ± 0.04 ^a^	3.23 ± 0.07 ^b^	2.25 ± 0.02 ^c^	1.23 ± 0.1 ^d^	1.16 ± 0.06 ^d^
C20:1	2.44 ± 0.02 ^b^	2.27 ± 0.04 ^c^	3.17 ± 0.04 ^a^	0.51 ± 0.03 ^d^	0.31 ± 0.03 ^e^	2.44 ± 0.02 ^a^	2.313 ± 0.04 ^b^	2.37 ± 0.06 ^b^	0.12 ± 0.07 ^d^	1.19 ± 0.11 ^c^	2.44 ± 0.02 ^c^	2.56 ± 0.05 ^b^	2.8 ± 0.01 ^a^	2.24 ± 0.07 ^d^	2.01 ± 0.08 ^e^	2.44 ± 0.02 ^c^	3.31 ± 0.06 ^a^	3.38 ± 0.01 ^a^	2.78 ± 0.02 ^b^	2.51 ± 0.1 ^c^
C21:0	0.26 ± 0.09 ^d^	0.38 ± 0.08 ^c^	0.5 ± 0.06 ^a^	0.47 ± 0.01 ^a^	0.42 ± 0.01 ^b^	0.26 ± 0.09 ^d^	0.47 ± 0.07 ^b^	0.45 ± 0.05 ^b^	0.36 ± 0.08 ^c^	0.54 ± 0.11 ^a^	0.26 ± 0.04 ^c^	0.39 ± 0.05 ^a^	0.15 ± 0.07 ^d^	0.28 ± 0.02 ^b^	0.19 ± 0.07 ^c^	0.26 ± 0.09 ^a^	0.15 ± 0.06 ^b^	0.24 ± 0.03 ^a^	0.23 ± 0.1 ^a^	0.11 ± 0.04 ^b^
C20:2n−6	0.52 ± 0.03 ^a^	0.35 ± 0.01 ^c^	0.34 ± 0.08 ^c^	0.48 ± 0.05 ^b^	0.45 ± 0.04 ^b^	0.52 ± 0.03 ^a^	0.373 ± 0.05 ^c^	0.31 ± 0.02 ^d^	0.4 ± 0.04 ^b^	0.42 ± 0.07 ^b^	0.52 ± 0.03 ^b^	0.29 ± 0.03 ^c^	0.55 ± 0.02 ^b^	0.56 ± 0.02 ^b^	0.71 ± 0.02 ^a^	0.52 ± 0.03 ^a^	0.45 ± 0.07 ^b^	0.36 ± 0.08 ^c^	0.53 ± 0.04 ^a^	0.58 ± 0.04 ^a^
C22:0	0.33 ± 0.04 ^d^	1.42 ± 0.08 ^a^	1.46 ± 0.01 ^a^	1.27 ± 0.05 ^b^	1.07 ± 0.04 ^c^	0.33 ± 0.04 ^d^	1.7 ± 0.07 ^a^	1.68 ± 0.08 ^a^	1.55 ± 0.07 ^b^	0.94 ± 0.06 ^c^	0.33 ± 0.04 ^a^	0.27 ± 0.07 ^a^	0.28 ± 0.08 ^a^	0.27 ± 0.07 ^a^	0.27 ± 0.07 ^a^	0.33 ± 0.04 ^a^	0.32 ± 0.1 ^a^	0.26 ± 0.01 ^b^	0.28 ± 0.09 ^a b^	0.27 ± 0.05 ^b^
C20:3n−6	5.63 ± 0.05 ^b^	5.93 ± 0.09 ^a^	5.59 ± 0.1 ^b^	5.57 ± 0.01 ^b^	5.17 ± 0.02 ^c^	5.63 ± 0.05 ^a^	5.14 ± 0.04 ^b^	5.04 ± 0.05 ^b^	4.72 ± 0.06 ^c^	3.63 ± 0.08 ^d^	5.63 ± 0.05 ^a^	5.3 ± 0.01 ^b^	5.25 ± 0.09 ^b^	4.98 ± 0.10 ^c^	4.26 ± 0.02 ^d^	5.63 ± 0.05 ^a^	5.55 ± 0.05 ^a^	5.56 ± 0.07 ^a^	4.55 ± 0.09 ^b^	4.38 ± 0.07 ^c^
C22:1	0.19 ± 0.01 ^d^	0.3 ± 0.01 ^b^	0.23 ± 0.01 ^c^	0.4 ± 0.04 ^a^	0.31 ± 0.04 ^b^	0.19 ± 0.01 ^c^	0.29 ± 0.03 ^b^	0.25 ± 0.03 ^b^	0.24 ± 0.04 ^b^	3.28 ± 0.03 ^a^	0.19 ± 0.01 ^e^	1.28 ± 0.08 ^d^	1.85 ± 0.03 ^a^	1.46 ± 0.07 ^c^	1.71 ± 0.02 ^b^	0.19 ± 0.01 ^b^	0.09 ± 0.09 ^c^	0.57 ± 0.06 ^a^	0.19 ± 0.09 ^b^	0.56 ± 0.01 ^a^
C20:3n−3	3.53 ± 0.05 ^a^	2.35 ± 0.03 ^b^	2.07 ± 0.02 ^c^	1.49 ± 0.1 ^d^	1.22 ± 0.01 ^e^	3.53 ± 0.05 ^a^	2.37 ± 0.06 ^c^	2.39 ± 0.02 ^c^	2.66 ± 0.03 ^b^	1.42 ± 0.06 ^d^	3.53 ± 0.05 ^a^	3.32 ± 0.02 ^b^	2.29 ± 0.03 ^c^	1.43 ± 0.10 ^d^	0.68 ± 0.06 ^e^	3.53 ± 0.05 ^a^	3.39 ± 0.02 ^b^	2.46 ± 0.05 ^c^	1.52 ± 0.07 ^d^	1.33 ± 0.03 ^e^
C20:4n−6	0.45 ± 0.03 ^a^	0.22 ± 0.05 ^b^	0.19 ± 0.09 ^b^	0.4 ± 0.05 ^a^	0.39 ± 0.01 ^a^	0.45 ± 0.03 ^a^	0.37 ± 0.09 ^a^	0.40 ± 0.02 ^a^	0.19 ± 0.1 ^b^	0.13 ± 0.04 ^b^	0.45 ± 0.03 ^a^	0.22 ± 0.01 ^b^	0.23 ± 0.08 ^b^	0.23 ± 0.05 ^b^	0.47 ± 0.03 ^a^	0.45 ± 0.03 ^a^	0.28 ± 0.07 ^c^	0.31 ± 0.09 ^b c^	0.39 ± 0.04 ^b^	0.21 ± 0.1 ^c^
C22:2n−6	2.74 ± 0.06 ^a^	2.03 ± 0.07 ^b^	1.98 ± 0.03 ^b^	1.43 ± 0.04 ^c^	1.18 ± 0.04 ^d^	2.74 ± 0.06 ^a^	1.43 ± 0.06 ^c^	2.17 ± 0.04 ^b^	2.27 ± 0.04 ^b^	1.12 ± 0.01 ^d^	2.74 ± 0.06 ^a^	2.34 ± 0.1 ^b^	2.14 ± 0.03 ^c^	2.08 ± 0.03 ^c^	1.77 ± 0.05 ^d^	2.74 ± 0.06 ^a^	2.57 ± 0.02 ^b^	2.36 ± 0.09 ^c^	1.91 ± 0.06 ^d^	1.35 ± 0.05 ^e^
C20:5n−3	0.38 ± 0.1 ^b^	0.62 ± 0.1 ^a^	0.63 ± 0.06 ^a^	0.61 ± 0.03 ^a^	0.58 ± 0.04 ^a^	0.38 ± 0.1 ^c^	0.63 ± 0.04 ^b^	0.71 ± 0.03 ^b^	0.65 ± 0.1 ^b^	1.16 ± 0.07 ^a^	0.38 ± 0.1 ^d^	0.47 ± 0.06 ^b^	0.48 ± 0.1 ^c^	0.44 ± 0.1	0.91 ± 0.08 ^a^	0.38 ± 0.1 ^c^	0.36 ± 0.01 ^c^	0.49 ± 0.01 ^b^	0.77 ± 0.02 ^a^	0.67 ± 0.07 ^a^
C22:3n−6	4.21 ± 0.1 ^d^	5.53 ± 0.06 ^c^	4.06 ± 0.04 ^e^	6.49 ± 0.01 ^a^	5.94 ± 0.08 ^b^	4.21 ± 0.1 ^d^	5.56 ± 0.1 ^c^	8.11 ± 0.07 ^a^	8.15 ± 0.02 ^a^	7.92 ± 0.06 ^b^	4.21 ± 0.1 ^a^	4.00 ± 0.04 ^b^	2.33 ± 0.06 ^d^	2.62 ± 0.05 ^c^	2.00 ± 0.04 ^e^	4.21 ± 0.1 ^a^	3.47 ± 0.09 ^c^	3.67 ± 0.01 ^b^	2.73 ± 0.02 ^d^	2.66 ± 0.06 ^d^
SFA	27.96 ± 0.23 ^e^	33.1 ± 0.14 ^d^	35.25 ± 0.16 ^c^	35.36 ± 0.25 ^b^	37.36 ± 0.27 ^a^	27.96 ± 0.23 ^e^	34.05 ± 0.19 ^d^	35 ± 0.11 ^c^	37.13 ± 0.16 ^b^	38.59 ± 0.18 ^a^	27.96 ± 0.23 ^e^	29.12 ± 0.11 ^d^	33.69 ± 0.14 ^c^	35.44 ± 0.23 ^b^	36.16 ± 0.18 ^a^	27.96 ± 0.23 ^e^	29.34 ± 0.18 ^d^	31.76 ± 0.18 ^c^	34.87 ± 0.19 ^b^	37.86 ± 0.15 ^a^
MUFA	20.98 ± 0.13 ^c^	19.84 ± 0.12 ^c^	22.71 ± 0.18 ^a^	21.67 ± 0.21 ^a^	21.25 ± 0.21 ^b^	20.98 ± 0.13 ^b^	20.997 ± 0.13 ^b^	21.46 ± 0.16 ^a^	20.74 ± 0.15 ^b^	21.21 ± 0.17 ^a^	20.98 ± 0.13 ^d^	22.74 ± 0.19 ^c^	23.13 ± 0.12 ^b^	22.85 ± 0.16 ^c^	24.47 ± 0.26 ^a^	20.98 ± 0.13 ^d^	23.69 ± 0.11 ^c^	24.1 ± 0.19 ^b^	25.56 ± 0.17 ^a^	25.73 ± 0.11 ^a^
PUFA	51.06 ± 0.24 ^a^	47.06 ± 0.22 ^b^	43.04 ± 0.28 ^c^	42.97 ± 0.29 ^d^	41.39 ± 0.35 ^e^	51.06 ± 0.24 ^a^	44.953 ± 0.13 ^b^	43.54 ± 0.23 ^c^	42.13 ± 0.14 ^d^	40.2 ± 0.22 ^d^	51.06 ± 0.24 ^a^	48.14 ± 0.14 ^b^	43.18 ± 0.22 ^c^	41.71 ± 0.19 ^d^	39.37 ± 0.28 ^e^	51.06 ± 0.24 ^a^	46.97 ± 0.21 ^b^	44.14 ± 0.12 ^c^	39.57 ± 0.18 ^d^	36.41 ± 0.22 ^e^

Note. Values were mean ± SD (n = 3). Values of EG and CG in a row with different superscript lowercase letters (a–e) differed significantly (*p* < 0.05).

**Table 6:** Regression equations for TVB-N and shelf-life of *Decapterus maruadsi* at different storage temperatures.

	Temperature/K	Regression Equations	Regression Coefficient (R^2)^	Reaction Rate Constant k (k)
EG	298.15	Y = 0.43903x + 1.73265	0.96108	0.43903
	310.15	Y = 1.98921x + 1.26075	0.93633	1.98921
CG	298.15	Y = 0.48708x + 1.82417	0.98093	0.48708
	310.15	Y = 2.20737x + 3.1455	0.93288	2.20737
	Storage temperature/K	Predicted value (h)	Actual value (h)	Relative error value (%)
EG	277.15	56.6365	54	4.60%
	310.15	12.5000	12	4.00%
GC	277.15	42.7086	40	6.35%
	310.15	9.4241	9	4.46%

## Data Availability

The data presented in this study are openly available in the article.
